# Immediate postoperative biomarkers characterize severity of primary graft dysfunction after heart transplantation

**DOI:** 10.1016/j.xjon.2026.101863

**Published:** 2026-05-13

**Authors:** Shreyas Kiran, Phoebe Miller, Isha Maniyar, Jashdeep Dhillon, Sarah Sullivan, Andrew Akcelik, Amy G. Fiedler, Jason Smith

**Affiliations:** a School of Medicine, University of California San Francisco, San Francisco, Calif; b Department of Surgery, University of California San Francisco, San Francisco, Calif; c Department of Cardiothoracic Surgery, University of California San Francisco, San Francisco, Calif

**Keywords:** primary graft dysfunction, heart transplantation, venoarterial carbon dioxide gap, blood lactate, biomarkers, hemodynamics, postoperative complications

## Abstract

**Objective:**

Primary graft dysfunction (PGD) remains the leading cause of early morbidity and mortality after orthotopic heart transplantation. Postoperative lactate and the venoarterial carbon dioxide gap (Pv-a CO_2_) are routinely measured indicators of hypoperfusion, yet their relationship to PGD severity is not well characterized. This study evaluated the association between immediate posttransplant (T0) and 24-hour (T24) biomarker values and the severity of PGD.

**Methods:**

We performed a single-center retrospective study of adult recipients of orthotopic heart transplantation (May 2020 to May 2023). PGD was defined according to International Society for Heart and Lung Transplantation criteria and categorized as none, moderate, or severe. Pv-a CO_2_ and serum lactate were collected at T0 for multivariable analysis and at T24 for descriptive and exploratory modeling. Patients receiving a simultaneous heart-lung transplant were excluded. Multivariable logistic regressions adjusted for age, body mass index, and sex.

**Results:**

Among 110 recipients, PGD occurred in 50 (45%) cases, including 39 moderate and 11 severe. At T0, both biomarkers demonstrated stepwise increases with PGD severity. Each 1-mm Hg increase in Pv-a CO_2_ was associated with greater odds of any PGD (odds ratio [OR], 1.53; 95% CI, 1.22-1.92), moderate PGD (OR, 1.48; 95% CI, 1.15-1.92), and severe PGD (OR, 2.07; 95% CI, 1.45-2.97). T0 lactate was independently associated with moderate PGD (OR, 1.21; 95% CI, 1.03-1.43). By 24 hours, Pv-a CO_2_ no longer independently differentiated PGD severity, whereas T24 lactate remained associated with moderate PGD (OR, 1.68; 95% CI, 1.13-2.49).

**Conclusions:**

Immediate posttransplant Pv-a CO_2_ is a strong discriminator of PGD severity and may serve as an early, objective adjunct to International Society for Heart and Lung Transplantation criteria. In contrast, lactate reflects both initial hypoperfusion and the subsequent trajectory of metabolic recovery, with persistent elevations at 24 hours indicating ongoing physiologic stress. Together, these readily available biomarkers provide complementary information that may improve early diagnostic stratification of PGD.

**Figure:**
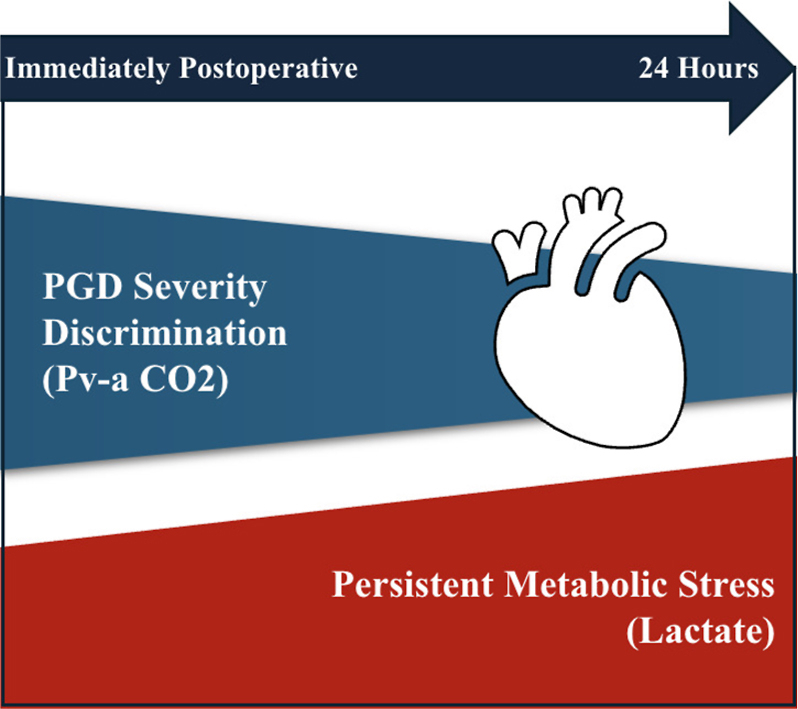
Postoperative biomarkers distinguish early PGD severity and persistent metabolic stress.

**Central Message**

Immediate postoperative venoarterial CO_2_ gap discriminates primary graft dysfunction severity after heart transplantation, and serum lactate reflects ongoing metabolic stress postoperatively.

**Perspective**

Early assessment of primary graft dysfunction severity is limited by delayed echocardiography and treatment-dependent diagnostic criteria. This study shows that routinely measured postoperative biomarkers can be adjuncts to current protocols. The venoarterial carbon dioxide gap discriminates graft dysfunction severity, whereas lactate reflects ongoing metabolic stress across the postoperative course.

Primary graft dysfunction (PGD) remains the leading cause of early morbidity and mortality after orthotopic heart transplantation (OHT).^1^, ^2^, ^3^, ^4^ The 2014 International Society for Heart and Lung Transplantation (ISHLT) consensus guidelines define PGD as ventricular dysfunction occurring within the first 24 hours after transplant, excluding secondary causes such as hyperacute rejection, pulmonary hypertension, and surgical complications. Left ventricular PGD includes both single- and biventricular failure, with mild, moderate, and severe classifications on the basis of metrics like ventricular function, inotropic support requirements, and need for mechanical circulatory support.^5^

Unfortunately, there are often challenges with applying these criteria in practice. Echocardiography in the immediate postoperative period, which is essential for grading according to ISHLT guidelines, is frequently not performed in the first 24 hours. Hemodynamic measurements can be confounded by vasoplegia or mechanical support.^6^ This can pose a challenge to distinguishing the severity of cardiac dysfunction from expected postbypass physiology. Practice patterns of initiating mechanical circulatory support also vary significantly across centers, which can introduce bias into treatment-based criteria for defining PGD.^7^ These limitations highlight the need for objective biomarkers of hemodynamic instability and tissue hypoperfusion that are available immediately after implantation and do not depend on clinician-driven intervention.^2^^,^^7^

Routinely measured physiologic markers have the potential to help better characterize the severity of graft dysfunction during the first 24 hours of the critical postimplantation window. Serum lactate indicates tissue hypoxia and inadequate perfusion, and venoarterial carbon dioxide gap (Pv-a CO_2_) reflects cardiac output and tissue perfusion adequacy.^8^^,^^9^ Elevations in both of these markers have been associated with low-flow states after cardiac surgery, although their associations with the severity of PGD after OHT has not been well characterized. The objective of this study was to characterize the association between immediate postoperative lactate and Pv-a CO_2_ values and severity of PGD.

## Methods

### Study Design and Patient Cohort

This was a retrospective analysis of a tertiary care center's cardiac transplant surgical database maintained at the University of California, San Francisco (UCSF). Patients were included if they had undergone a heart transplant at UCSF between May 2020 and May 2023. The electronic medical records of individual patients were reviewed and relevant data collected. This study was approved by the Committee on Human Research at UCSF (institutional review board 24-41355 approved February 26, 2024). Consent was not required.

### Primary Outcome

The primary outcome was the presence and severity of PGD in the immediate postoperative period after transplantation, categorized as none, moderate, or severe according to adapted ISHLT criteria. PGD severity was determined using postoperative hemodynamics and inotropic support requirements as detailed herein. The primary exposures of interest were the immediate postoperative (T0) Pv-a CO_2_ (mm Hg) and serum lactate concentration (mmol/L), obtained immediately after documented procedure end-time. Biomarker values at 24 hours (T24) were also collected for descriptive analyses but were not included as predictors in multivariable models.

### Primary Graft Dysfunction

PGD was classified according to the 2020 ISHLT criteria: moderate PGD was defined as having a vasoactive-inotropic score >10 and a mean arterial pressure <70 mm Hg without mechanical circulatory support.^10^ Severe PGD was defined as requiring mechanical circulatory support in the postsurgical period (eg, venoarterial extracorporeal membrane oxygenation [ECMO] or percutaneous ventricular-assist device) within 24 hours of transplantation. Recipients who met neither criterion were classified as having no PGD. Given the increased need for postoperative ECMO in patients undergoing simultaneous lung transplant, these patients were excluded from our sample.

### Statistical Analysis

Descriptive statistics were applied to summarize and tabulate data and logistic regression analysis was applied to adjust for significant variables. Cross-tabulations were used to investigate associations between both preoperative and intraoperative patient characteristics and PGD. χ^2^ tests and Mann-Whitney *U* tests were applied to categorical and continuous variables, respectively. Binomial multivariate logistic regression to examine the associations between each biomarker measured at T0 for (1) any PGD versus none, (2) moderate PGD versus none, and (3) severe PGD versus none. A multinomial regression was used including clinical risk factors for PGD and variables noted to be significantly associated with PGD in our univariate analysis. Backwards selection was used to fit the model and decrease co-linearity between variables. Odds ratios (ORs) are reported per 1-unit increase in the biomarker including 95% CIs. A 2-sided alpha of 0.05 was used to determine statistical significance for all analyses. Data analyses were performed using the pandas library (version 2.2.2) in Python; Python, version 3.12.4.

## Results

### Demographics

Between May 2020 and May 2023, 110 adults underwent OHT. PGD occurred in 50 (45.5%) OHT recipients. Of those cases, 39 (35.5%) were classified as having moderate and 11 (10.0%) were severe PGD. Age (49.96 ± 14.76 years), sex distribution (27.3% female), body mass index (27.33 ± 5.5 kg/m^2^), race/ethnicity, and overall comorbidity burden did not differ significantly across the 3 groups (Table 1).

**Table 1:** Univariate analysis of demographics and clinical characteristics

Variable	No PGD (n = 60)	Moderate PGD (n = 39)	Severe PGD (n = 11)	Total (n = 110)	*P* value
Age at transplant, y	49.95 ± 14.78	50.59 ± 14.78	47.82 ± 15.85	49.96 ± 14.76	.86
Sex, female	16 (26.7%)	10 (25.6%)	4 (36.4%)	30 (27.3%)	.77
Race/ethnicity					.89
White	18 (30%)	17 (43.6%)	4 (36.4%)	39 (35.5%)	
Hispanic or Latino	17 (28.3%)	8 (20.5%)	3 (27.3%)	28 (25.5%)	
Black or African American	10 (16.7%)	7 (17.9%)	3 (27.3%)	20 (18.2%)	
Asian	8 (13.3%)	3 (7.7%)	1 (9.1%)	12 (10.9%)	
Native American or Alaska Native	0 (0.0%)	1 (2.6%)	0 (0.0%)	1 (0.9%)	
Native Hawaiian/Pacific Islander	1 (1.7%)	0 (0.0%)	0 (0.0%)	1 (0.9%)	
Other	5 (8.3%)	2 (5.1%)	0 (0.0%)	7 (6.4%)	
BMI	26.91 ± 5.48	27.73 ± 5.37	28.24 ± 6.32	27.33 ± 5.50	.65
BSA	1.94 ± 0.25	1.99 ± 0.29	2.07 ± 0.41	1.97 ± 0.28	.33
Simultaneous transplants					.93
0	52 (86.7%)	34 (87.2%)	10 (90.9%)	96 (87.3%)	
1	8 (13.3%)	5 (12.8%)	1 (9.1%)	14 (12.7%)	
DCD vs. DBD					.70
DBD	58 (96.7%)	36 (92.3%)	11 (100.0%)	105 (95.5%)	
DCD	2 (3.3%)	2 (5.1%)	0 (0.0%)	4 (3.6%)	
Comorbidities					
COPD	1 (1.7%)	1 (2.6%)	1 (9.1%)	3 (2.7%)	.45
Hypertension	27 (45.0%)	19 (48.7%)	3 (27.3%)	49 (44.5%)	.27
Hyperlipidemia	13 (21.7%)	12 (30.8%)	1 (9.1%)	26 (23.6%)	.24
Diabetes	18 (30.0%)	6 (15.4%)	1 (9.1%)	25 (22.7%)	.08
Renal disease	19 (31.7%)	9 (23.1%)	3 (27.3%)	31 (28.2%)	.54
Dialysis	3 (5.0%)	1 (2.6%)	1 (9.1%)	5 (4.5%)	.68
Smoking history	12 (20.0%)	7 (17.9%)	1 (9.1%)	20 (18.2%)	.58
Previous chest surgery	22 (36.7%)	20 (51.3%)	6 (54.5%)	48 (43.6%)	.37

*PGD*, Primary graft dysfunction; *BMI*, body mass index; *BSA*, body surface area; *DCD*, donor by cardiac death; *DBD*, donor by brain death; *COPD*, chronic obstructive pulmonary disease.

### Perioperative Characteristics

The majority (95.5%, n = 105) of transplanted grafts were donated after brain death, whereas only 3.6% (n = 4) were donated after cardiac death. A small portion (12.7%, n = 14) of patients underwent an additional simultaneous transplant (Table 1). All patients who left the operating room on mechanical circulatory support were specifically initiated on ECMO.

### Univariate Analysis

At the immediate postoperative time point (T0), mean Pv-aCO_2_ increased with the degree of PGD severity. In recipients without PGD the mean Pv-aCO_2_ was 5.62 ± 2.54 mm Hg. In those with moderate PGD, the mean Pv-aCO_2_ was 9.05 ± 5.31 mm Hg and in severe PGD the mean was 14.45 ± 5.39 mm Hg (*P* < .01). Serum lactate followed a similar pattern, increasing from a mean lactate of 4.69 ± 3.07 mmol/L in recipients without PGD to 6.75 ± 4.22 mmol/L in moderate PGD and 8.24 ± 4.07 mmol/L in severe PGD (*P* < .01) (Table 2).

**Table 2:** Univariate analysis of postoperative variables

Biomarker	No PGD (n = 60)	Moderate PGD (n = 39)	Severe PGD (n = 11)	Total (n = 110)	*P* value
T0					
Mixed venous PCO_2_	45.42 ± 5.26	48.28 ± 7.84	50.09 ± 9.57	46.90 ± 6.91	**.03**
Arterial PCO_2_	39.80 ± 4.59	39.23 ± 6.43	35.85 ± 6.09	39.18 ± 5.54	.07
Pv-a CO_2_	5.62 ± 2.54	9.05 ± 5.31	14.45 ± 5.39	7.72 ± 4.86	**<.01**
Serum lactate	4.69 ± 3.07	6.75 ± 4.22	8.24 ± 4.07	5.75 ± 3.81	**<.01**
T24					
Mixed venous PCO_2_	42.53 ± 4.79	44.03 ± 5.31	49.67 ± 4.39	43.69 ± 5.29	**<.01**
Arterial PCO_2_	37.59 ± 4.44	38.22 ± 4.59	38.89 ± 6.88	37.93 ± 4.71	.67
Pv-a CO_2_	5.32 ± 4.41	5.81 ± 3.07	10.78 ± 4.52	5.95 ± 4.24	**<.01**
Serum lactate	1.69 ± 0.80	2.29 ± 1.46	1.88 ± 0.55	1.92 ± 1.09	**.03**

Bolded *P* values indicate statistical significance (*P* < .05).

*PGD*, Primary graft dysfunction; *T0*, time point with a laboratory value immediately after procedure end time; *PCO*_*2*_, partial pressure of carbon dioxide; *Pv-a CO*_*2*_, venoarterial carbon dioxide gap; *T24*, time point with a laboratory value closest to 24 hours after procedure end time.

At 24 hours (T24), the observed Pv-aCO_2_ gradient persisted: 5.32 ± 4.41 mm Hg in recipients without PGD, 5.81 ± 3.07 mm Hg in moderate PGD, and 10.78 ± 4.52 mm Hg in severe PGD (*P* < .01). Serum lactate followed a similar gradient: 1.69 ± 0.80 without PGD, 2.29 ± 1.46 in moderate PGD, and 1.88 ± 0.55 in severe PGD (*P* = .03) (Table 2).

### Multivariate Analysis

After adjustment for age at transplant, body mass index, and sex, each 1-mm Hg increase in T0 Pv-a CO_2_ was associated with 53% greater odds of any PGD (OR, 1.53; 95% CI ,1.22-1.92; *P* < .01) compared with no PGD. Each 1-mmol/L increase in T0 lactate was associated with 23% greater odds of any PGD (OR, 1.23; 95% CI, 1.04-1.44; *P* = .01 (Table 3). In severity-specific models, T0 Pv-a CO_2_ remained independently associated with both moderate PGD (OR, 1.48; 95% CI, 1.15-1.92, *P* < .01) and severe PGD (OR, 2.07; 95% CI, 1.45-2.97, *P* < .01), whereas serum lactate corresponded only with moderate PGD (OR, 1.21; 95% CI, 1.03-1.43, *P* = .02) (Tables 4 and 5).

**Table 3:** Multivariate regression of immediately and 24 hours postoperative CO_2_ gap and lactate with any PGD versus no PGD

Variable	T0			T24		
	Odds ratio	95% CI	*P* value	Odds ratio	95% CI	*P* value
Pv-a CO_2_	1.53	(1.22-1.92)	**<.01**	1.09	(0.91-1.31)	.36
Serum lactate	1.23	(1.04-1.44)	**.01**	1.62	(1.10-2.38)	**.02**
Age at transplant	1.01	(0.98-1.04)	.65	1.01	(0.98-1.04)	.58
BMI	1.02	(0.94-1.11)	.62	1.02	(0.95- 1.10)	.59
Sex (female)	1.01	(0.33-3.09)	.99	1.35	(0.55-3.30)	.51

Bolded *P* values indicate statistical significance (*P* < .05).

*CO*_*2*_, Carbon dioxide; *PGD*, primary graft dysfunction; *T0*, time point with a laboratory value immediately after procedure end time; *T24*, time point with a laboratory value closest to 24 hours after procedure end time; *Pv-a CO*_*2*_, venoarterial carbon dioxide gap; *BMI*, body mass index.

**Table 4:** Multivariate regression of immediately and 24 hours postoperative CO_2_ gap and lactate with moderate PGD versus no PGD

Variable	T0			T24		
	Odds ratio	95% CI	*P* value	Odds ratio	95% CI	*P* value
Pv-a CO_2_	1.48	(1.15-1.92)	**<.01**	1.02	(0.91-1.14)	.76
Serum lactate	1.21	(1.03-1.43)	**.02**	1.68	(1.13-2.49)	**.01**
Age at transplant	1.01	(0.98-1.04)	.67	1.01	(0.98-1.04)	.47
BMI	1.01	(0.93-1.10)	.86	1.02	(0.94-1.10)	.61
Sex (female)	1.00	(0.32-3.12)	1.0	1.10	(0.40-3.06)	.85

Bolded *P* values indicate statistical significance (*P* < .05).

*CO*_*2*_, Carbon dioxide; *PGD*, primary graft dysfunction; *T0*, time point with a laboratory value immediately after procedure end time; *T24*, time point with a laboratory value closest to 24 hours after procedure end time; *Pv-a CO*_*2*_, venoarterial carbon dioxide gap; *BMI*, body mass index.

**Table 5:** Multivariate regression of immediately and 24 hours postoperative CO_2_ gap and lactate with severe PGD versus no PGD

Variable	T0			T24		
	Odds ratio	95% CI	*P*-value	Odds ratio	95% CI	*P* value
Pv-a CO_2_	2.07	(1.45-2.97)	**<.01**	1.22	(0.83-1.79)	.31
Serum lactate	1.12	(0.80-1.55)	.51	1.26	(0.61-2.60)	.53
Age at transplant	1.00	(0.94-1.05)	.85	1.00	(0.94-1.07)	.91
BMI	1.09	(0.86-1.38)	.50	1.03	(0.89-1.18)	.72
Sex (female)	0.74	(0.08-6.91)	.79	2.83	(0.57-14.07)	.20

Bolded *P* values indicate statistical significance (*P* < .05).

*CO*_*2*_, Carbon dioxide; *PGD*, primary graft dysfunction; *T0*, time point with a laboratory value immediately after procedure end time; *T24*, time point with a laboratory value closest to 24 hours after procedure end time; *Pv-a CO*_*2*_, venoarterial carbon dioxide gap; *BMI*, body mass index.

At 24 hours posttransplant, T24 Pv-a CO_2_ was not associated with any PGD (OR, 1.09; 95% CI, 0.91-1.31; *P* = .36). In contrast, each 1-mmol/L increase in T24 lactate remained significantly associated with any PGD (OR, 1.62; 95% CI, 1.10-2.38; *P* = .02) (Table 3). In severity-specific models, T24 Pv-a CO_2_ continued to show no independent association with moderate PGD (OR, 1.02; 95% CI, 0.91-1.14, *P* = .76) or severe PGD (OR 1.22; 95% CI, 0.83-1.79, *P* = .31). However, T24 lactate remained significantly associated with moderate PGD (OR, 1.68; 95% CI, 1.13-2.49, *P* = .01) and lost significance with severe PGD (OR, 1.26, 95% CI 0.61-2.60, *P* = .53).

## Discussion

In this single-center cohort, immediate postoperative Pv-a CO_2_ and serum lactate were independently associated with PGD. Previous work has established both metabolic derangements as markers of hypoperfusion after cardiac surgery.^8^^,^^11^ Intraoperative lactic acidosis has been suggested as a potential diagnostic marker for PGD, and posttransplant lactic acidosis has been shown to be associated with PGD and increased inotropic support.^7^^,^^9^ Our study offers a focused analysis of 2 biomarkers that can serve as adjuncts in our definition of PGD.

Our work extends these observations by demonstrating the association between postoperative lactate, Pv-a CO_2_ and the severity of PGD. The increase in Pv-a CO_2_ from 5.6 mm Hg in patients without PGD to 14.5 mm Hg in severe PGD underscores its sensitivity to the low-output physiology of early graft failure. Previous work has suggested that Pv-a CO_2_ directly reflects acute hemodynamic dysfunction, and we are not aware of previous studies to demonstrate this relationship across ISHLT PGD severity in the heart transplant population.^8^ Although the greater venous carbon dioxide (CO_2_) burden is intuitive and physiologically consistent with decreased cardiac output, the gradient in Pv-a CO_2_ gap across PGD severity strata is important. Intensive care unit protocols typically initiate inotropes or mechanical circulatory support on the basis of invasive hemodynamics and clinical judgment.^3^^,^^5^^,^^7^ Our results suggest that an immediately posttransplant Pv-a CO_2_ value could be an objective indicator of more advanced physiologic compromise even before other metrics are collected or signs of shock manifest. It is important to note that both serum lactate and Pv-a CO_2_ are affected by the timing of ECMO initiation or the use of an oxygenator. However, our T0 laboratory values were drawn immediately postoperatively while the patient was still in the operating room, leaving minimal time for any initiated mechanical support to substantially alter serum lactate or CO_2_ clearance. This approach provides a readily available complement to the hemodynamic criteria (right atrial pressure >15 mm Hg, pulmonary capillary wedge pressure >20 mm Hg, cardiac index <2.0 L/min/m^2^) detailed in the ISHLT consensus definition, particularly in settings where early invasive monitoring may be delayed or confounded by vasoplegia.^5^

In contrast, the association between serum lactate and PGD plateaued as the dysfunction progressed from moderate to severe. This could be explained by the initiation of ECMO. The relationship between lactate and inotropic support is well-established, with previous work showing a strong correlation (*P* < .02) between peak lactate levels and maximal inotropic dosing in heart transplant recipients.^12^ This interaction may confound the interpretation of lactate as a pure marker of graft dysfunction in the severe PGD category where inotropic requirements are greatest.

At 24 hours postoperatively, biomarker behavior reflects both underlying graft function and early postoperative management. In our cohort, Pv-a CO_2_ had largely converged by 24 hours and lost significance across PGD strata. This is consistent with early ECMO initiation, high-dose inotropic support, and rapid improvement in CO_2_ clearance. This suggests that Pv-a CO_2_ is most informative in the immediate posttransplant period as an early hemodynamic marker and offers less insight into the progression or resolution of PGD once perfusion has been corrected. Lactate remained significantly associated with moderate PGD at 24 hours, indicating that incomplete lactate clearance over the first 24 hours still reflects persistent metabolic stress in early graft dysfunction. Elevated lactate was associated with greater odds of both moderate and severe PGD at 24 hours compared to immediately postoperatively. We anticipate that their association with severe PGD at 24 hours is partially blunted due to early initiation of mechanical circulatory support. This pattern is consistent with previous findings that lactate peaks early and normalization over 24 hours is influenced by inotropic intensity rather than perfusion alone.^12^ By 24 hours, however, persistent metabolic stress and incomplete recovery and lactate more clearly characterizes this physiologic severity.

These findings should be interpreted in the context of ongoing research to build models that characterize and predict PGD. Traditional risk prediction tools like the RADIAL score (based on recipient right atrial pressure, age, diabetes, inotrope dependence, donor age, and ischemic time) have shown poor discrimination in contemporary cohorts (area under the curve, 0.53), prompting development of machine learning models and exploration of novel biomarkers.^7^ Currently research has focused on donor biomarkers like troponin, B-type natriuretic peptide, procalcitonin, and more recently donor C-type lectin domain family 4 member C, but these can require specialized assays or sample collection before and during procurement.^7^ Pv-a CO_2_ and serum lactate are routinely monitored during and after transplant. Predictive biomarkers identify risk before graft dysfunction is diagnosed, while our approach offers diagnostic stratification once PGD is clinically suspected.

There are a few primary limitations in our study. First, the single-center retrospective design limits generalizability; however, it enabled granular and nuanced data collection of fluctuating parameters that are often not captured in national databases. This level of physiologic detail was essential for evaluating early postoperative Pv-a CO_2_ and lactate trajectories, and it would not have been feasible in larger datasets. Second, we were unable to classify mild PGD or categorize right ventricular and biventricular PGD subtypes because of the lack of consistent postoperative echocardiographic data.^5^ This limitation reflects a broader challenge in the field of a lack of routine echocardiography within the first 24 hours after transplant, making mild PGD difficult to reliably identify across centers and underscoring the need for more clinically practical PGD definitions. Third, both Pv-a CO_2_ and lactate reflect broader perfusion inadequacy and may be elevated as the result of factors independent of PGD, including hypovolemia, vasoplegia, and hepatic or renal impairment. These biomarkers should therefore be interpreted as adjuncts to clinical assessment and not as isolated diagnostic markers. In addition, a broader panel with potentially relevant biomarkers including serum bicarbonate, pH, and troponin represents an important direction for future investigation.

## Conclusions

In this single-center cohort of 110 recipients of heart transplant, we demonstrated that both Pv-a CO_2_ gap and serum lactate measured immediately posttransplant are independently associated with PGD severity, with Pv-a CO_2_ showing the strongest gradient across severity strata. Our findings suggest that incorporating postoperative Pv-a CO_2_ measurements into intensive care unit protocols could complement existing ISHLT hemodynamic criteria and facilitate earlier, more objective risk stratification, particularly in settings where early comprehensive invasive monitoring may be delayed or confounded by vasoplegia.

## Conflict of Interest Statement

The authors reported no conflicts of interest.

The *Journal* policy requires editors and reviewers to disclose conflicts of interest and to decline handling or reviewing manuscripts for which they may have a conflict of interest. The editors and reviewers of this article have no conflicts of interest.

## References

[1] Singh S.S.A., Dalzell J.R., Berry C., Al-Attar N. (2019). Primary graft dysfunction after heart transplantation: a thorn amongst the roses. Heart Fail Rev.

[2] Sicim H., Tam W.S.V., Tang P.C. (2024). Primary graft dysfunction in heart transplantation: the challenge to survival. J Cardiothorac Surg.

[3] Hull T.D., Crowley J.C., Villavicencio M.A., D’Alessandro D.A. (2021). Primary graft dysfunction in heart transplantation: how to recognize it, when to institute extracorporeal membrane oxygenation, and outcomes. J Thorac Cardiovasc Surg Open.

[4] Buchan T.A., Moayedi Y., Truby L.K. (2021). Incidence and impact of primary graft dysfunction in adult heart transplant recipients: a systematic review and meta-analysis. J Heart Lung Transplant.

[5] Kobashigawa J., Zuckermann A., Macdonald P. (2014). Report from a consensus conference on primary graft dysfunction after cardiac transplantation. J Heart Lung Transplant.

[6] Byrne J. (2004). Risk factors and outcomes for “vasoplegia syndrome” following cardiac transplantation. Eur J Cardiothorac Surg.

[7] Dhingra N.K., Foroutan F., Truby L.K. (2026). When bad things happen to good hearts prediction and management of primary graft dysfunction. Can J Cardiol.

[8] Ariza M., Gothard J.W.W., Macnaughton P., Hooper J., Morgan C.J., Evans T.W. (1991). Blood lactate and mixed venous-arterial PCO z gradient as indices of poor peripheral perfusion following cardiopulmonary bypass surgery. Intensive Care Med.

[9] The American Association for Thoracic Surgery | AATS. Rising lactate. https://www.aats.org/resources/rising-lactate-after-orthotopi-8742.

[10] Udesen N.L.J., Beske R.P., Hassager C. (2025). Microaxial flow pump hemodynamic and metabolic effects in infarct-related cardiogenic shock: a substudy of the DanGer shock randomized clinical trial. JAMA Cardiol.

[11] Chemmalakuzhy J., Costanzo M.R., Meyer P. (2001). Hypotension, acidosis, and vasodilatation syndrome post–heart transplant: prognostic variables and outcomes. J Heart Lung Transplant.

[12] Mohacsi P., Pedrazzini G., Tanner H., Tschanz H., Hullin R., Carrel T. (2002). Lactic acidosis following heart transplantation: a common phenomenon?. Eur J Heart Fail.

